# Supplementary material for: Dynamic transcriptomic profiles of zebrafish gills in response to zinc supplementation
Source: BMC Genomics. 2010 Oct 11;11:553. doi: 10.1186/1471-2164-11-553 (PMC3091702; doi:10.1186/1471-2164-11-553)
Supplement: Additional file 2 — Interactive Direct Interaction Network representing the molecular interactions between zinc, copper, iron, calcium and proteins encoded by transcripts changed by zinc supplementation. Mini web-site containing index.html and hyperlinked pages in subdirectory describing a Direct Interaction Network automatically generated based on curated interactions contained within the proprietary PathwayArchitect database. Ovals represent proteins and the circles symbolize metal ions. Objects are coloured by their abundance in zebrafish at the time-point they were significantly different from the control is a scale from -4 fold (dark green) to +4 fold (dark red). Where significant differences were found at more than one time-point, the colour overlay shows expression at the first instance. Dark blue squares denote 'binding', and light blue squares 'expression'; green squares stand for 'regulation', green diamonds for 'metabolism', and green circles for 'promoter binding'. Arrow heads indicate directionality of the interaction where annotated. All nodes and edges can be further interrogated by selecting the relative area of the image. [file 1471-2164-11-553-S2.zip › PathwayArchitect Zn xs DIN/875041.html]

# REGULATION:

|  |  |
| --- | --- |
| Type | REGULATION |
| Effect | Positive |


---

|  |  |
| --- | --- |
| Score | 0 |


---

|  |  |
| --- | --- |
| Reference Count | 4 |


---

|  |  |
| --- | --- |
| Mechanism | Unknown |


---

|  |  |
| --- | --- |
| Reference:0 || Sentence | "HNPP is associated with a 1.5-Mb deletion in chromosome 17p11.2-12 and may result from reduced expression of the PMP22 gene." |
| PMID | 7804455 |
| Year | 1994 |
| Species | Human |
| Journal | Curr Opin Neurol |
| RefScore | 1 |
| Source | PArchNLP |
  |
|  |  |


---

|  |  |
| --- | --- |
 Reference:1 || Sentence | "A 1.5-Mb deletion in chromosome 17p11.2-p12 has been associated with HNPP." |
| PMID | 7825607 |
| Year | 1995 |
| Species | Human |
| Journal | Am J Hum Genet |
| RefScore | 1 |
| Source | PArchNLP |
  ||  |  |


---

|  |  |
| --- | --- |
 Reference:2 || Sentence | "We now show that transgenic mice expressing antisense PMP22 RNA exhibit modestly reduced levels of PMP22 together with a phenotype that is reminiscent of hereditary neuropathy with liability to pressure palsies (HNPP), a human disease caused by a 1.5-Mb deletion of a chromosome 17 region that contains the PMP22 gene." |
| PMID | 9143558 |
| Year | 1997 |
| Species | Mouse |
|  | Human |
| Journal | Mol Cell Neurosci |
| RefScore | 2 |
| Source | PArchNLP |
  ||  |  |


---

|  |  |
| --- | --- |
 Reference:3 || Sentence | "The HNPP locus maps to chromosome 17p11.2-12 and is associated with a 1.5-Mb deletion." |
| PMID | 10586225 |
| Year | 1999 |
| Species | Human |
| Journal | Ann N Y Acad Sci |
| RefScore | 2 |
| Source | PArchNLP |
  |


---

|  |  |
| --- | --- |
